# Management of Chronic Atrophic Candidiasis (Denture Stomatitis)—A Narrative Review

**DOI:** 10.3390/ijerph20043029

**Published:** 2023-02-09

**Authors:** Eman Abuhajar, Kamran Ali, Gulraiz Zulfiqar, Khalifa Al Ansari, Hina Zafar Raja, Shaza Bishti, Lamyia Anweigi

**Affiliations:** 1 College of Dental Medicine, QU Health, Qatar University, Doha 2713, Qatar; 2 Jinnah Hospital, Lahore 54550, Pakistan; 3 Hamad Medical Corporation, Doha 3050, Qatar; 4 CMH Lahore Medical College, Institute of Dentistry, Lahore 54810, Pakistan; 5 Department of Prosthodontics and Biomaterials, Aachen University Hospital, 52074 Aachen, Germany

**Keywords:** antifungal drugs, *Candida albicans*, denture stomatitis, oral candidiasis, phytomedicine, sore mouth

## Abstract

One of the most common oral diseases affecting people wearing dentures is chronic atrophic candidiasis or denture stomatitis (DS). The aim of the paper is to provide an update on the pathogenesis, presentation, and management of DS in general dental practice settings. A comprehensive review of the literature published in the last ten years was undertaken using multiple databases, including PubMed via MEDLINE, EMBASE, and Scopus. The eligible articles were analyzed to identify evidence-based strategies for the management of DS. Despite its multifactorial nature, the leading cause of DS is the development of oral *Candida albicans* biofilm, which is facilitated by poor oral and denture hygiene, long-term denture wear, ill-fitting dentures, and the porosity of the acrylic resin in the dentures. DS affects between 17 and 75% of the population wearing dentures, with a slight predominance in elderly females. The mucosal denture surfaces and posterior tongue are the common sites of DS, and the affected areas exhibit erythema, the swelling of the palatal mucosa and edema. Oral and denture hygiene protocols, adjusting or re-fabricating poorly adapting dentures, smoking cessation, avoiding nocturnal denture wear, and the administration of topical or systemic antifungals are the mainstay of management. Alternate treatments such as microwave disinfection, phytomedicine, photodynamic therapy, and incorporation of antifungals and nanoparticles into denture resins are being evaluated for the treatment of DS but require further evidence before routine use in clinical practice. In summary, DS is the most common oral inflammatory lesion experienced by denture wearers. Most patients with DS can be managed in general dental practice settings. Effective management by general dental practitioners may be supported by a thorough understanding of the pathogenesis, the recognition of the clinical presentation, and an awareness of contemporary treatment strategies.

## 1. Introduction

Chronic atrophic candidiasis, commonly referred to as denture stomatitis (DS), is the most prevalent multifactorial, chronic inflammatory oral condition amongst denture wearers [1,2]. It affects edentulous people who wear complete or partial dentures, as well as those who use intraoral removable orthodontic appliances and obturators [3].

DS most commonly involves the palate and is more likely to be observed in patients with acrylic dentures than prostheses fabricated using other materials or in other locations [4,5]. As elderly people are more likely to use dentures, the condition is far more prevalent in older populations. However, DS is also seen in healthy, younger people who wear dentures [6].

### 1.1. Pathogenesis

The precise pathogenies of DS are not known, but infection with *Candida* is most likely to be associated with mucosal trauma induced by ill-fitting dentures, sub-optimal oral hygiene, the nocturnal wearing of dentures, and xerostomia [1,7]. The fitting surface of dentures provides a protected environment for the entrapment of yeast cells, which are able to colonize the irregularities in the denture-base and denture-relining materials [8]. This is more likely to occur in patients with other risk factors, such as poor oral hygiene and the continuous wearing of dentures.

Local risk factors associated with denture stomatitis are dry mouth, denture age, local trauma induced by an ill-fitting or poorly fabricated dentures, poor denture hygiene, microorganisms, continuous and nocturnal denture wearing, smoking, carbohydrate-rich diets, acidic salivary pH, and sensitivity to denture materials [7,9,10,11,12,13,14,15,16].

The oral microbiome of denture wearers is less diverse than those of fully dentate patients and may demonstrate higher rates of oral *Candida* carriage [17]. *Candida* displays dimorphism and can exist both in yeast and hyphal forms. The yeast form is observed in the carriage of oral *Candida* as commensals, while the hyphal form is associated with tissue invasion and disease, i.e., Candidiasis. Dentures provide a microenvironment that encourages *Candida* colonization [14]. The longstanding use of dentures in patients with poor oral hygiene allows the dental biofilm (plaque) to colonize the surface of the prosthesis and the mucosal surfaces in contact with the denture base [18,19].

Saliva has a dual role in Candidal adhesion to polymethylmethacrylate (PMMA). Saliva exhibits a physical cleansing effect and consists of antimicrobial components, such as lysozymes, immunoglobulins, glycoproteins, lactoferrin, and peroxidase. These constituents interact with *Candida* species and reduce their adherence and colonization on oral mucosal surfaces. However, some salivary proteins, such as mucins and statherins, can act as receptors for the nanoproteins present in Candidal cell walls and promote their adhesion [20]. Decreased salivary flow underneath the fitting surface of dentures further promotes the adhesion of *Candida* to the denture base and adjacent mucosal surfaces [21,22]. Ultimately, the *Candida* may develop into the hyphal form, infiltrate the mucosal tissue, and cause inflammation, which manifests clinically as DS.

The presence of a prosthesis is a prerequisite, and poor oral hygiene and the continuous use of dentures are the most significant risk factors for developing DS. *Candida albicans.*

Oral mucosal trauma is also a risk factor for DS in susceptible patients and may result from ill-fitting or rocking full or partial dentures [23]. Both histological and microbiological analyses of mucosal tissue have shown that trauma has a significant role in the development of this condition [24]. Research suggests that trauma results in an inflammatory reaction, which creates a favorable environment for *C. albicans* to invade the tissues and initiate an inflammatory reaction [25]. Candidal growth has also been associated with the use of soft denture liners, often used to improve the fit of dentures. Soft liners are prone to deterioration, increasing their roughness and enhancing the risk of Candidal colonization [26]. Regarding prosthesis-related factors, an allergy in the form of contact mucositis may occur due to the presence of resin monomers, hydroquinone peroxide, dimethyl-p -toluidine, or methacrylate in the denture. Contact allergies are more common with cold- or auto-cured resins than with heat-cured denture-base materials due to the higher monomer content of the former [27]

Systemic risk factors include debilitating physiological aging, poorly controlled diabetes mellitus, xerostomia, radiation therapy, neoplasm, nutritional deficiency, immunocompromised conditions (immunosuppression, immunodeficiency, as with HIV infection, and radiation therapy for the head and neck region), the long-term use of corticosteroids and antibiotics, and hematological disorders. These factors might contribute by compromising an individual’s resistance to combat the disease [7,9,14,15,21,22]. Recently, De Souza et al. [28] reported that individuals with low socioeconomic status are more likely to develop DS due to poor access to routine medical care. An association relationship between DS and other diseases, such as pneumonia and bacterial endocarditis, is also reported [3,19,28].

### 1.2. Classification, Prevalence, and Clinical Presentation

The first recognized classification of DS was proposed by Newton in 1962 [29], which still remains in use and groups DS as follows:

Type I: manifests as localized mucosal inflammation induced by trauma.

**Figure 1:**
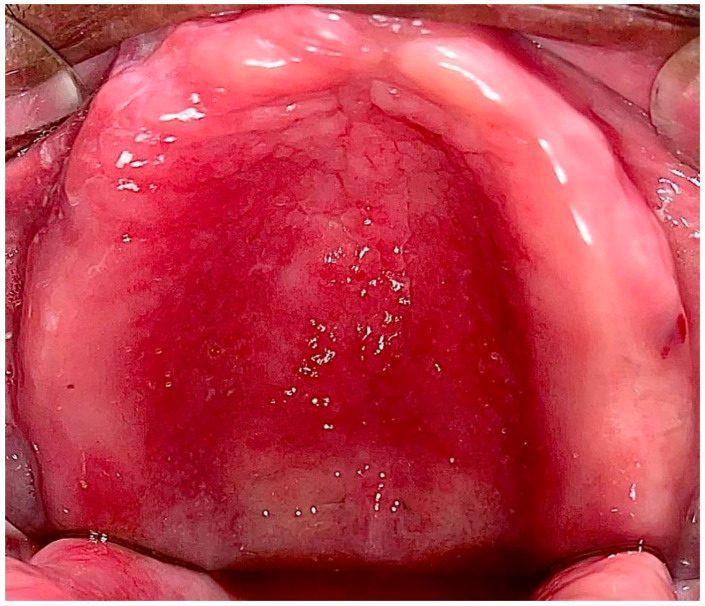
Diffuse involvement of the denture-bearing mucosa in a patient with an upper full denture.

**Figure 2:**
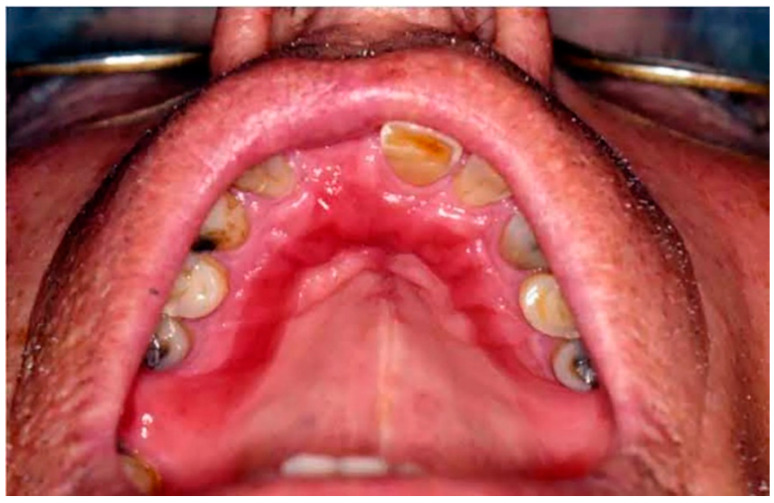
Diffuse involvement of the denture-bearing mucosa in a patient with an upper partial denture.

**Figure 3:**
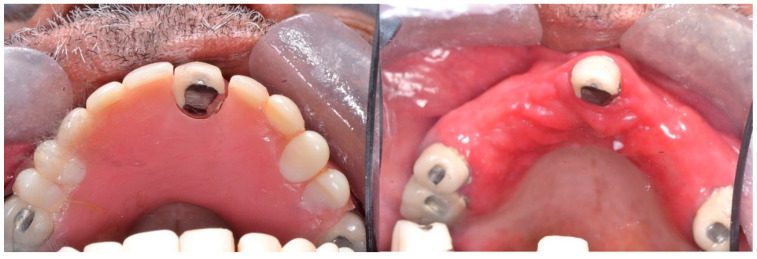
Diffuse involvement of the denture-bearing mucosa mirroring the partial denture base.

Type II: Diffuse involvement of the denture-bearing mucosa (Figure 1, Figure 2 and Figure 3).

**Figure 4:**
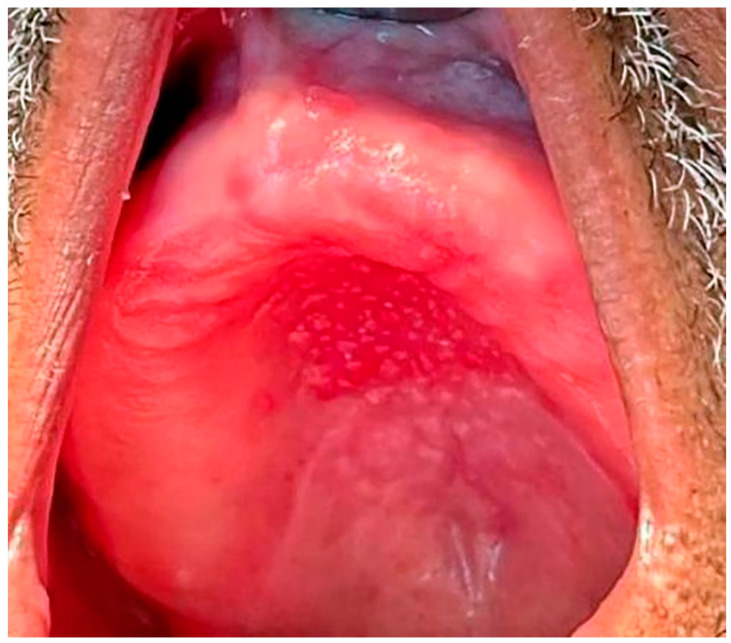
Inflammatory Papillary Hyperplasia in a patient with an upper full denture, the denture-bearing mucosa shows a granular appearance.

Type III: Additionally known as inflammatory papillary hyperplasia, the denture-bearing mucosa shows a granular appearance (Figure 4).

Although Newton’s classification is clinically useful for grading the hyperemia of denture-bearing mucosa, it does not accurately reflect the severity and extent of the disease [30].

A classification system has been proposed by Barbeau et al. [31] that subdivides Newton types into additional categories based on the extent of the lesions. The classification put forth by Schwartz et al. [32] subdivides Newton types I, II, and III based on the severity of inflammation using a score ranging from zero to six. Recently, Neppelenbroek et al. [30] proposed a modified Newton classification based on three clinical features: (a) appearance (I, II, and III), (b) the degree of inflammation of the palatal denture-bearing mucosa per quadrant, and (c) the degree of erythema (slight or intense redness). These scores for the three criteria are combined to rank the severity of DS using a total of 24 points.

One in every three denture wearers suffers from DS, the most common oral mucosal lesion associated with removable dentures [28]. The prevalence of DS ranges from 60 to 70% in denture wearers who exhibit clinical signs and symptoms [18,21]; however, this percentage may be up to 75% if asymptomatic patients are included [33].

The common sites of development of DS are the palate, tonsillar area, maxillary ridge, and posterior tongue [34,35]. In addition, Sartawi et al. [21] reported multiple small papillary nodules and papillary proliferations on the labial surface and labial mucosa, respectively. The conditions are rare in mandibular denture-bearing areas due to the washing action of saliva to clear the biofilm accumulation [36].

DS manifests clinically as variable mucosal erythema with or without dispersed petechiae in the areas covered by the denture base. The affected mucosa often has a palpable shape that mirrors the overlying denture. Despite the angry appearance of the affected mucosa due to marked erythema, most patients do not report any soreness. However, a minority of patients may report irritation of the affected oral mucosa, ulcerations, or a burning sensation, dysgeusia, dysphagia, and halitosis. Concurrent lesions commonly associated with DS include angular cheilitis, atrophic glossitis, and pseudomembranous or hyperplastic candidiasis [7,19,21]. The presence of prolonged inflammation may enhance the patient’s chance of developing cardiovascular disease, diabetes, pulmonary disease, and lead to the progression of systemic infections. [19,28].

Given that DS is commonly encountered in general dental practice settings and a plethora of research on this topic has been published in recent years, this article aims to provide an update on the management of DS.

## 2. Methods

A scoping search was initially carried out to identify and estimate the volume of primary studies on DS.

### 2.1. Eligibility Criteria

The scoping search was used to map key search terms and develop the inclusion and exclusion criteria, as summarized below.

The inclusion criteria are as follows:

- Type of study: Randomized controlled clinical trials (RCTs) and systematic reviews; (based on RCTs) on the management of DS in human subjects;
- Period: Studies published from January 2012 to December 2022;
- Language: Studies published in English were considered.

The exclusion criteria are as follows:

o Animal or cadaveric studies;
o In vitro studies;
o Studies conducted before 2012;
o Studies published in languages other than English;
o Unpublished studies.

### 2.2. Information Sources

All types of peer-reviewed research on DS that were published from January 2012 to December 2022. The literature from relevant databases, including Embase, Scopus, and Web of Science, were included in the review. In addition, a hand search of relevant publications was performed using Google Scholar with the reference lists of the retrieved papers from the above databases.

### 2.3. Search Strategy

The search was conducted using key search items, including “*Denture stomatitis*,” and/or “Denture-associated erythematous stomatitis,” and/or “Chronic atrophic candidiasis” and/or “Denture-induced stomatitis,” and/or “Inflammatory papillary hyperplasia,” and/or “Denture sore mouth,” and/or “Denture,” and/or “Candidiasis,” and/or “Candida-associated denture stomatitis.”

### 2.4. Data Screening

All of the identified articles were imported into EndNote^®^, version X9 (Calrivate plc, London, UK), Clarivate Analytics. After the removal of duplicates, two reviewers independently screened the titles and abstracts. A full-text screening was conducted for the studies that adhered to the inclusion criteria. Any disagreements were resolved by consensus.

## 3. Results

### Study Selection

**Figure 5:**
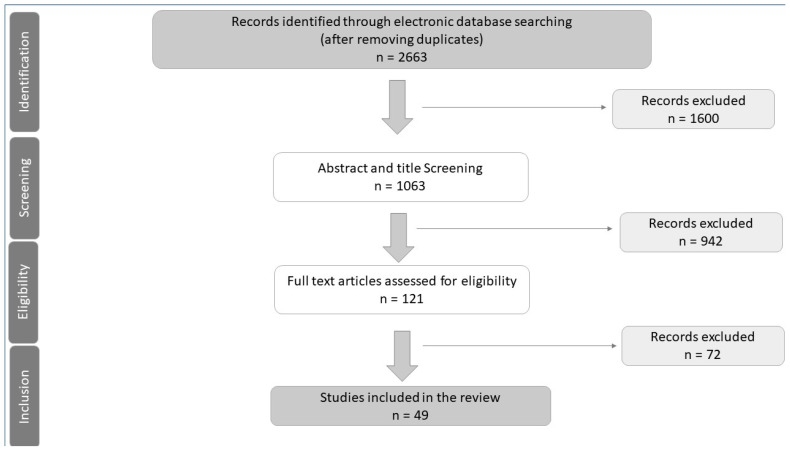
PRISMA Flow Chart.

The results of the search and study selection are shown in the PRISMA flow chart in Figure 5. The total number of studies identified was 2663 (2657 from the databases and six reports from the grey literature). After the removal of duplicates, 1062 articles were included in the title and abstract screening. Of these, 935 from the databases and six from the grey literature were excluded, and 121 articles were considered for a full-text screening. Finally, 49 articles were selected for inclusion, including 34 randomized controlled clinical trials and 15 systematic reviews (Table 1).

The data in the selected studies were collated to dissect the existing literature relating to the management of DS. The studies focused on a range of treatments for DS, including antifungal agents, lasers, photodynamic therapy, microwave radiation, phytomedicine using natural products, and antimicrobial agents. Of the 34 RCTs, 31 were evaluated in the 15 systematic reviews [37,38,39,40,41,42,43,44,45,46,47,48,49,50] included in this study. The main findings of the systematic reviews based on RCTs are summarized in Table 1.

Only three RCTs were not evaluated by any of the 15 systematic reviews included in this study. All three RCTs focused on the efficacy of different antimicrobial agents to reduce the *Candida* colonization of dentures but showed a moderate-to-high risk of bias. Auon et al. (2015) reported that the overnight immersion of dentures in 0.12% chlorhexidine digluconate and 0.2% cetylpyridinium chloride is effective in reducing the *Candida* colonization of acrylic dentures [51]. Badaró et al. (2020) [52] reported an RCT that showed an efficacy of 0.25% sodium hypochlorite as a disinfectant for denture cleaning. Finally, Procópio et al. (2022) [53] reported on the benefits of incorporating minimum inhibitory concentrations of antimicrobials (nystatin or chlorhexidine diacetate) in denture liners.

## 4. Discussion

**Figure 6:**
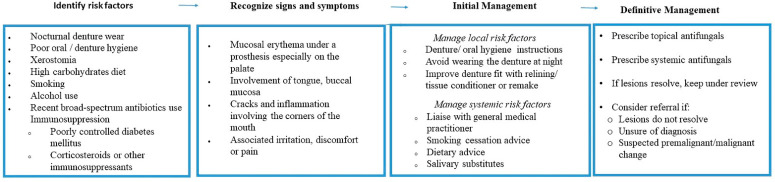
A schematic management of DS.

General dental practitioners may find it challenging to assimilate the vast amount of information on the management strategies for DS and apply it effectively in their clinical practice [21]. A systematic approach to the management of DS requires dentists to identify and control the local and systemic risk factors associated with DS. Patients should be advised on meticulous plaque control and the avoidance of the nocturnal wearing of dentures, usually for eight hours per day [21,54]. Existing dentures must be evaluated for any potential flaws relating to fit and texture and managed appropriately, including the possibility of replacement dentures. Addressing the underlying risk factors, oral hygiene measures, and appropriate antifungal therapy is likely to be effective in resolving the majority of cases. A schematic management of DS is summarized in Figure 6.

### Identification of Risk Factors

The local and systemic risk factors for DS must be evaluated comprehensively with a thorough medical, dental, and social history and eliminated/controlled where appropriate.

(a) Oral and denture hygiene protocol

Meticulous oral hygiene, including prosthesis care, is important in achieving the resolution of DS [55]. Riberio et al. [2] proposed a hygiene protocol that has shown to be effective in reducing the inflammation associated with DS and involves the following:

- Brushing the hard palate for 2 min, three times a day with soft toothbrush bristles and water;
- During the day, brush the dentures for 2 min, three times a day, with a denture brush and neutral, non-abrasive liquid soap;
- Before going to bed at night, soak the dentures in 150 mL of 0.25–0.5% sodium hypochlorite for 10–20 min, followed by the overnight storage of the dentures in fresh, clean water at room temperature.

Although mechanical cleaning is a quick and straightforward method to control plaque, it can result in the wear of the denture base materials, leading to flaws on the surface of the dentures that encourage the growth of biofilms and pigmentation [56,57]. In contrast, there are several options for using chemical cleansers, including hypochlorite, peroxides, and enzymes, with sodium hypochlorite being the preferable agent. Denture wearers prefer chemical denture cleansers because they are easy to use, affordable, and they have been effective in reducing biofilm formation [58,59]. However, repeatedly soaking the dentures in hypochlorite may lead to discoloration of the dentures. The use of chlorhexidine gel with anti-discoloration systems (e.g., Curasept 0.5%) can be used to reduce *Candida* colonization on PMMA resin with minimal discoloration and limited changes to the mechanical properties of PMMA [60].

High-frequency electromagnetic radiation, such as microwaves, can either cause cell death by changing the structure of the cell and the permeability of its membrane or can cause cell death through an interaction between the electromagnetic field created by the microwaves and the molecules of the cell [47]. Microwave ovens have been recommended as a simple and cost-effective method for cleaning prostheses. It can be an effective alternative to antifungal medications for the treatment of DS [47].

Da Costa et al. [37] reported that microwave disinfection was more effective than topical miconazole alone. It produced mycological and clinical results comparable to an overnight soak in a solution of 0.2% chlorhexidine and 0.02% sodium hypochlorite. Although there is no standardized procedure for cleaning and disinfecting dentures in a microwave, a disinfection protocol at 650 W for 3 min once a week for two consecutive weeks has been recommended to prevent and treat DS with minimal possible denture damage [37]. However, such claims should be confirmed by further long-term clinical studies.

(b) Antifungal therapy

Antifungals are often prescribed in conjunction with other measures to treat established cases of DS. Polyenes (nystatin and amphotericin B) and Azoles, which are categorized into triazoles (itraconazole and fluconazole) and imidazoles (clotrimazole, ketoconazole, isoconazole, miconazole, and tioconazole) are the two classes of antifungals that are most frequently used for DS treatment [54]. Azole antifungals potentiate the effects of oral anticoagulant warfarin, and this needs to be discussed with the patient’s physician to adjust the dosage of the drug appropriately. The usual duration of antifungal therapy is up to 14 days.

Topical antifungal medications, such as nystatin, which are available as dry powders, lozenges (pastilles), and suspensions, are effective against most *Candida* species.

- Dry powder forms: Approximately ⅛ teaspoonful of dry powder is added to 4 ounces of water and stirred thoroughly. The medicine can then be used as a mouthwash/gargle;
- Lozenges (pastilles): Need to be held in the mouth and allowed to dissolve slowly over 15–30 min;
- 1–2 Tablets or lozenges may be used 3–5 times/day;
- Oral suspensions: Hold 4 to 6 milliliters (mL) in the mouth for a few minutes, then swish it around, and gargle before swallowing it. Repeat four times/day.

Patients with full or partial dentures may need to soak their dentures nightly in a nystatin oral suspension to eliminate the fungus from the dentures. In rare cases, when this does not eliminate the fungus, it may be necessary to have new dentures made.

Miconazole is available as an oral gel (24 mg/mL). A small pea-sized amount (2.5 mls) of the gel is applied over the affected area 2–4 times/day. It can also be applied to the fitting surface of an upper denture before placing the denture in the mouth. Repeat this 3–4 times daily.

Systemic therapy has been recommended for DS in cases where topical medications are ineffective, such as in individuals who need special care or in patients with systemic comorbidities, such as diabetes or immunosuppression [54,61]. Systemic antifungal drugs that have undergone the most extensive research and are the most effective are fluconazole and itraconazole [54].

A study by Vidya et al. [62] demonstrated that incorporating fluconazole and ketoconazole antifungal agents into tissue conditioners positively inhibited *Candida albicans* growth. Similarly, Shaikh et al. [63], through their systematic review, reported that the addition of nystatin to tissue conditioners proved beneficial.

Although numerous antifungal medications are available, DS frequently recurs following antifungal therapy, which necessitates retreatment with antifungals. The persistence of *Candida* biofilms on the mucosa and inert prosthetic surfaces could contribute to DS relapse. Common impediments to complete recovery include reduced patient compliance, immunodeficiency, and drug resistance associated with frequent daily dosages and the unjustified use of antifungals over time. Regardless of the choice of DS therapy, factors related to the prostheses, such as poor oral and denture hygiene, the nocturnal wear of dentures, and failure to replace old faulty prostheses, contribute to lesion relapse [28,64].

It is also worth mentioning that although fluconazole is one of the most popular systemic antifungals, it only has a fungistatic action against *C. albicans*, which may result in fluconazole resistance after prolonged use. [65] There is emerging evidence that the combination of fluconazole with other drugs, such as myriocin [66] (a fungus-derived antibiotic), may result in a synergistic effect that may potentiate the effects of fluconazole against *C. albicans*. However, such interventions need further evidence before routine clinical use.

(c) Nanoparticles and antimicrobials

Silver (Ag), titanium dioxide (TiO_2_), zinc oxide (ZnO), and zirconium dioxide (ZrO_2_) nanoparticles have been shown to exhibit antimicrobial properties. Silver nanoparticles (AgNPs) demonstrate antimicrobial activity due to their ability to damage cell membranes and could be added to PMMA in a concentration of 3–3.5 wt% to reduce *C. albicans* colonization [67]. Similarly, the addition of TiO_2_ and halloysite clay in PMMA can also reduce the colonization of the dentures by *C. albicans* [68]

The application of an antimicrobial coating on denture surfaces has been suggested to inhibit *Candida albicans* adhesion by creating smooth denture surfaces [69]; however, the coating obliterates surface details and tissue adaptability, which may have an impact on denture retention. Additionally, mechanical and chemical denture hygiene may damage the coating, resulting in a rougher surface with a higher propensity for microbial colonization [14].

Conflicting results are reported regarding the applications of nanoparticles or antimicrobials for the treatment of DS. Bajunaid [70] recommended the use of various antimicrobial and protein-repellent agents to prevent *Candida albicans* from adhering to acrylic dentures. However, the addition of these antimicrobials can compromise the mechanical properties of acrylic commensurate with the concentration of antimicrobials. On the contrary, An et al. [70], in their systematic review, reported that there was insufficient evidence to support the effectiveness of adding antimicrobial agents to acrylic. Similar findings are reported by other studies [71].

(d) Photodynamic therapy

Photodynamic therapy (PDT), also termed photodynamic antimicrobial chemotherapy, photo radiation therapy, and photo chemotherapy, has recently gained popularity as a potential antifungal treatment modality [72]. The most popular photosensitizers used in dentistry are methylene blue, toluidine blue ortho, and indocyanine green.

A review of recent publications has shown that PDT was just as effective as conventional antifungal medications, particularly nystatin, in the clinical remission of DS and considerably reducing the colony-forming units (CFU/mL) of *Candida* species from dentures [18,35,39,42,43]. However, the recurrence of DS following PDT has also been reported [18]. Since PDT is recommended as an alternative to conventional antifungal medication, more rigorous research with longer follow-up is required before their inclusion in clinical practice. One study by Yousef et al. [73] recommends PDT combined with low-level laser therapy and miconazole gel for the efficient treatment of *Candida*-induced DS, with a higher cure rate and lower recurrence.

(e) Phytomedicine

Phytomedicine, which has existed since the dawn of human civilization, can be defined as herbal or natural products with therapeutic and medicinal values [74]. Natural products are said to be effective in treating DS because they have improved the clinical and microbiological aspects of the condition [45].

Natural products such as curcumin, propolis, green tea, clove and cinnamon oils, chitosan, garlic, ginger, *Zataria multiflora*, Artemisia, *Schinus terebinthifolius raddi*, *Uncaria tomentosa*, *Pelargonium graveolens*, *Equisetum giganteum*, *Melaleuca alternifolia*, *Punica granatum*, *Ricinus communis*, henna, thymoquinone, and aloe-vera have been used in the treatment of DS (Silveira et al., 2021). These products can be applied topically or as loaded emulgels. Previous studies have suggested that the incorporation of natural products into the acrylic resin denture base material might be effective in preventing *Candida albicans* adhesion. Compared to traditional antifungals, these products have a better taste, comparable efficacy, fewer side effects, and higher patient satisfaction [45,65,74]

Several natural products have been studied for the treatment of DS. Despite the encouraging results reported in the literature when using natural products, more clinical research is needed to furnish appropriate scientific evidence to support the use of natural products for DS management [45].

## 5. Conclusions

DS represents the most common inflammatory reaction in denture wearers. Although DS has a multifactorial etiology, infection with oral *Candida albicans* is the most recognized etiological agent. Observing oral and denture hygiene regimens, adjusting or re-fabricating poorly adapting dentures, smoking cessation, avoiding nocturnal denture wear, and the administration of topical or systemic antifungal drugs are recommended for effective healing of the inflammatory lesions of DS. Alternate treatments, such as microwave disinfection, phytomedicine, photodynamic therapy, and the incorporation of antifungals and nanoparticles into denture resins are widely reported in the literature but further evidence is required before such alternate treatment options can be recommended routinely.

## Figures and Tables

**Table 1:** Interventions for the Management of Denture Stomatitis.

Author/Year	Intervention(s)	Number of RCTs Included	Main Findings	Risk of Bias
da Costa et al., 2020 [37]	Microwave	5	Microwave disinfection is equally effective as 0.2% chlorhexidine, 0.02% sodium hypochlorite, and topical nystatin (100,000 IU/mL), and superior to topical miconazole	Moderate
Davoudi 2018 [35]	Low-level laser therapy (LLLT) or photodynamic therapy (PDT	6	LLLT has a significant role in the clinical treatment of DS PDT and showed similar results to conventional antifungal therapies	Low–moderate
Emami E 2014 [38]	Efficacy of antifungal therapy with alternative methods	14	Disinfection methods could be considered as an adjunct to antifungal medications; no statistically significant difference between antifungals and disinfection for clinical and microbiological outcomes	Moderate–high
Firoozi et al., 2021 [39]	Antimicrobial Photodynamic therapy (aPDT) compared to Nystatin	3	aPDT may be effective in reducing *Candida* colony count and treating DS but is not superior to nystatin	Moderate
Hilgert et al., 2016 [40]	Any agent or procedure to treat DS	35	Nystatin and disinfecting agents can be an effective treatment for DS	High
Lyu et al., 2016 [41]	Comparison of nystatin with other antifungal agents	11	Nystatin pastille alone or pastille with suspension is more effective than suspension alone; prolonged treatment duration (4 weeks) can increase the efficacy of nystatin; Fluconazole superior in infants, children, or HIV patients	Moderate–high
Rai et al., 2022 [42]	Compared topical nystatin to other antifungal agents or placebo	24	Equal efficacy of 100,000 IU of nystatin suspension and six sessions of PDT for the treatment of DS	Moderate–high
Roomaney 2021 [43]	Photodynamic therapy	5	PDT comparable to systemic and topical antifungals	Low–moderate
Shui et al., 2021 [44]	Phytotherapy	19	Phytomedicines had fewer side effects and more patient satisfaction than antifungals or disinfectants; no statistical difference between propolis and miconazole for clinical and microbiological parameters	High
Silveira et al., 2021 [45]	Natural products (Propolis, Green tea, Ginger, Zataria multiflora, chitosan, garlic, Artemisia, Schinus terebinthifolius Raddi, Uncaria tomentosa, Punica granatum, and Ricinus communis)	14	Natural products showed similar efficacy and safety when compared with nystatin or miconazole	High
Skupien JA 2013 [46]	Nystatin (500,000 units) and Sodium hypochlorite 0.5%	7	Sodium hypochlorite 0.5% can disinfect denture liners & tissue conditioners; incorporation of nystatin is also beneficial	High
Sousa et al., 2021 [47]	Microwave disinfection	4	Efficient as an antifungal therapy	High
Verhaeghe et al., 2020 [48]	Overnight storage conditions	3	Cleaning dentures before overnight storage reduces *C. albicans*; overnight dry storage could reduce *C. albicans* colonization	Low
Vila-Nova TEL et al., 2022 [49]	Photodynamic therapy (PDT)	4	Photodynamic therapy is effective in improving the efficacy of antifungals	Low to moderate
Zhang et al. 2016 [50]	Miconazole	17	Miconazole oral gel may be more effective than other formulations with regard to long-term results	Moderate to high

## Data Availability

Data is contained within the article.
